# Sequence Alignment between *TRIM33* Gene and Human Noncoding RNAs: A Potential Explanation for Paraneoplastic Dermatomyositis

**DOI:** 10.3390/jpm14060628

**Published:** 2024-06-13

**Authors:** Rossella Talotta

**Affiliations:** Rheumatology Unit, Department of Clinical and Experimental Medicine, University Hospital “Gaetano Martino”, 98124 Messina, Italy; rtalotta@unime.it

**Keywords:** bioinformatics, cancer, dermatomyositis, ncRNAs, TIF1γ, *TRIM33*

## Abstract

Background: This computational analysis investigated sequence complementarities between the *TRIM33* gene and human noncoding (nc)RNAs and characterized their interactions in the context of paraneoplastic dermatomyositis. Methods: *TRIM33* FASTA sequence (NCBI Reference Sequence: NC_000001.11) was used for BLASTN analysis against Human GRCh38 in the Ensembl.org database. Retrieved ncRNAs showing hits to *TRIM33* were searched in the GeneCards.org database and further analyzed through RNAInter, QmRLFS-finder, Spliceator, and NcPath enrichment analysis. Results: A total of 100 hits were found, involving the lncRNAs NNT-AS1, MKLN1-AS, LINC01206, and PAXBP1-AS1, whose dysregulation has been reported in either cancer or dermatomyositis. Additionally, the lncRNAs NNT-AS1 and PAXBP1-AS1 may interact with microRNA-142-3p, reducing its expression and increasing that of *TRIM33*. Sequence complementarity affected only *TRIM33* intron 1, possibly resulting in alternatively spliced isoforms of TIF1γ with increased immunogenicity. The results also revealed nucleotide alignment between *TRIM33* and the gene regulatory elements of 28 ncRNA genes involved in immune pathways. Conclusions: This pivotal study demonstrates sequence complementarity between *TRIM33* and human ncRNAs dysregulated in cancer and dermatomyositis. This scenario may lead to the overproduction of more immunogenic TIF1γ variants in tumors and the stimulation of autoimmunity. Further experimental analyses using targeted methods such as Western blot or Chip-Seq are required to confirm these data.

## 1. Introduction

Idiopathic inflammatory myopathies (IIMs) consist of a heterogeneous spectrum of rare diseases that affect skeletal muscle and result in chronic inflammation and destruction of muscle fibers. IIMs include juvenile and adult dermatomyositis (DM), polymyositis (PM), immune-mediated necrotizing myositis (IMNM), inclusion body myositis (IBM), antisynthetase syndrome (ASyS), and myositides that overlap with other diagnoses such as systemic lupus erythematosus or mixed connective tissue disease [1,2]. Dermatomyositis (DM) is an IIM characterized by the simultaneous involvement of skeletal muscle and skin. As a result of the deposition of immune complexes on the endothelium of skeletal muscle, patients diagnosed with DM usually develop perifascial atrophy, which is clinically reflected by symmetric proximal muscle weakness [3,4]. In addition, the precipitation of immune complexes in dermal vessels and complement-mediated cell lysis along the dermo–epidermal interface are responsible for cutaneous manifestations such as photosensitive erythema on the neck, back, and shoulders, heliotrope rash, Gottron’s papules, and telangiectasia [5]. Furthermore, DM patients may develop pulmonary, gastrointestinal, and cardiac manifestations, including interstitial pneumonitis, cardiac arrhythmias, and esophageal dysmotility [6].

DM is the major IIM phenotype for which a clear association with cancer has been documented. The reported incidence of cancer in adult DM patients is estimated to be approximately 9% to 32% [7]. A cancer diagnosis may occur after or before the onset of DM, but usually the interval between the two conditions is no more than three years [2,7]. Given the close temporal relationship, DM may be considered a paraneoplastic phenomenon. Interestingly, more than 80% of adult patients with cancer-associated DM are characterized by serum positivity of autoantibodies that recognize a 155 kDa nuclear protein known as transcription intermediary factor 1 gamma (TIF1γ) [8]. Moreover, anti-TIF1γ-seropositive DM patients have a higher risk of severe disease, flagellar erythema, V-neck sign, and overall lower survival [3], while interstitial lung disease, Raynaud’s phenomenon, calcinosis, and joint involvement are less common in anti-TIF1γ-positive patients [9,10].

TIF1γ belongs to the triple motif (TRIM) superfamily and is encoded by the gene *TRIM33* located on the short arm of the chromosome 1 [11]. The protein appears to participate in cell mitosis and differentiation, DNA repair, transcriptional control, and translational modifications [10]. Its dual role as a tumor suppressor or inducer has been described in numerous cancer studies [10,12,13] and somatic mutations of the *TRIM33* gene have been reported in patients with paraneoplastic DM [14,15].

It is likely that the dysregulated expression of TIF1γ in the tumor microenvironment may contribute to the loss of immune tolerance and the formation of cross-reactive autoantibodies with the subsequent development of DM [11]. The latter event could be triggered by the immunological effects of this mediator on innate and acquired immune cells [12,16]. This hypothesis is supported by the observation that the fluctuation of anti-TIF1γ antibody titers may follow the progression of underlying malignancy or DM disease activity [17].

Research shows that cancer cells undergo transcriptomic changes that can lead to up- or down-regulation of genes involved in cell growth and differentiation [18]. This phenomenon can also generate neoepitopes that can be presented via major histocompatibility complex (MHC) class I or II [19]. Transcriptional activity is a highly dynamic process and is regulated by several mechanisms, including alternative splicing or polyadenylation and the expression of noncoding RNAs (ncRNAs). Indeed, several studies have shown imbalanced ncRNA transcription in cells from humans with tumors or DM [20,21,22]. However, it is not known whether TIF1γ expression can be affected by the dysregulated production of ncRNAs, which can occur in both cancer and DM patients. Altered cross-talk between ncRNAs and coding genes, such as *TRIM33*, may take place as an early event in malignant cells and lead to the activation of an immunological cascade resulting in the appearance of DM. Therefore, the aim of this work was to investigate possible interactions between the *TRIM33* gene and human ncRNAs based on sequence complementarities and RNA interactome analysis in silico and to correlate these results with the panorama of existing data on ncRNA profiling in cancer and DM.

## 2. Materials and Methods

### 2.1. Identification of Human ncRNAs Aligning to TRIM33 Gene Sequence

To investigate any nucleotide alignment between *TRIM33* and human ncRNAs, the FASTA sequence of the *TRIM33* gene, available at https://www.ncbi.nlm.nih.gov/nuccore/NC_000001.11?report=fasta&from=114392790&to=114511203&strand=true, accessed on 3 February 2023 (NCBI Reference Sequence: NC_000001.11), was used for BLASTN analysis against Human GRCh38 by consulting the Ensembl.org database (Ensembl ncRNA genes; https://www.ensembl.org/index.html accessed on 3 February 2023) [23]. Specifically, the input sequence consisted of the reverse complementary strand of the *TRIM33* gene with a length of 118414 base pairs (from 114392790 to 114511203), excluding the flanking 5′ and 3′ untranslated regions (UTRs).

For the analysis of the Ensembl.org database, the preconfigured normal sensitivity setting was chosen, which allows the exclusion of short sequences and distant homologies and does not limit the search to closer matches only. The default options (general options, scoring options, filtering and masking options) were the following: maximum number of hits to be reported: 100; maximum E-value for reported alignments: 10; maximum HSPs per hit: 100; match/mismatch score: 1,-3; opening and extension gap penalties: 2; low complexity region filter and query sequences with RepeatMasker filter: applied.

### 2.2. In Silico Characterization of Human ncRNAs Aligning to TRIM33 Gene Sequence

The detected ncRNAs that had hits for *TRIM33* were searched in the GeneCards.org database (https://www.genecards.org accessed on 16 February 2023) [24] and Genome Aggregation Database (gnomAD; https://gnomad.broadinstitute.org accessed on 16 February 2023) [25] to characterize their nature, tissue expression, subcellular localization, and known associated diseases. Furthermore, the exact genomic location showing hits for *TRIM33* was searched by consulting the Ensembl.org database [23], and the interaction with DNA and other ncRNAs was analyzed by using the tools RNAInter [26] and Quantitative Model of R-loop Forming Sequence (RLFS) finder (QmRLFS-finder) [27]. Specifically, RNAInter analysis was carried out by using the following parameters: keyword: ‘*TRIM33*’; interaction-type: ‘RR’; species: ‘homo sapiens’; method: ‘all’; category: ‘mRNA’; score: ‘0–1’. 

In addition, Spliceator was used to predict *TRIM33* splice sites in intronic regions aligning with ncRNAs [28].

Finally, NcPath enrichment analysis (http://ncpath.pianlab.cn/#/Home accessed on 18 March 2023) was carried out to characterize the biological pathways associated with ncRNAs of interest.

## 3. Results

### 3.1. Human ncRNAs Aligning with the TRIM33 Gene

By consulting the Ensembl.org database, a total of 100 hits between human ncRNAs and the *TRIM33* gene sequence were found (Table S1). In detail, the *TRIM33* gene aligned with 97 transcripts of long noncoding (lnc)RNA genes, 2 transcripts of uncategorized genes (*ENSG00000286456* and *ENSG00000269842*) and 1 pseudogene (*MFSD14CP*) with overall mean ± standard deviation (SD) nucleotide length of 352.3 ± 110.0, mean ± SD score of 548.2 ± 129.8, and mean ± SD percentage of identity (%ID) of 95.1 ± 2.6%. Complementary sequences to *TRIM33* gene were predicted in 2 or more transcripts of the same genes in the following cases: *ENSG00000286679* (13 hits); *PAXBP1-AS1* (8 hits); *XACT* (6 hits); *ENSG00000287277* and *LINC01551* (4 hits); *AQP4-AS1* and *HELLPAR* (3 hits); *ENSG00000273448*, *ENSG00000284294*, *ENSG00000286535*, *ENSG00000286797*, *LINC00824*, *LINC01116*, and *MKLN1-AS* (2 hits). Importantly, none of the hits matched the nucleotide sequence of *TRIM33* gene exons, as they were all located at intronic sites (Table S1).

When available, tissue expression was ubiquitous for most of the ncRNAs found, but in many cases the expression in the nervous system, intestine, and testis predominated. Most ncRNAs were not characterized for their subcellular localization; those for which this information was available were reported to localize in the nucleus (n°12), the nucleolus (n°6), the cytosol (n°3), and the extracellular compartment (n°7).

### 3.2. Human Diseases Associated with ncRNAs Aligning with the TRIM33 Gene

According to GeneCards.org database, ten ncRNA genes aligning with *TRIM33* nucleotide sequence have known associations with human disease, including cancer (Table 1). NcRNA genes whose polymorphic variants have been associated with cancer risk included *NNT-AS1, LINC01116*, and *SILC1*, which matched the *TRIM33* gene with a score of 903 (%ID 91.8%), 577 (%ID 98.7%), and 466 (%ID 97%), respectively.

**Figure 1:**
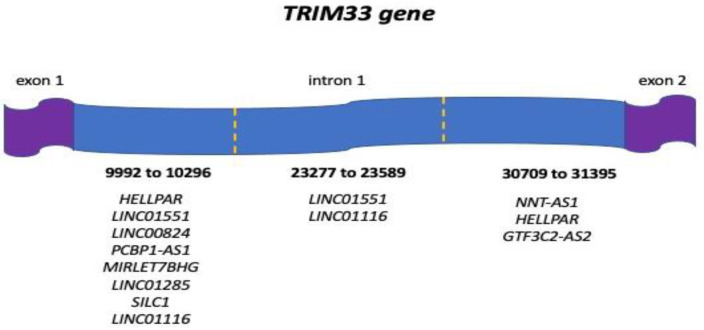
Aligned sequence location on the *TRIM33* gene of the 10 ncRNAs associated with cancer and other human diseases.

In detail, the matches on the *TRIM33* gene involved 3 different sites of intron 1 that could bind more than one ncRNA simultaneously according to the Ensembl.org analysis (Figure 1). Importantly, according to the GnomAD database, the hits did not involve nucleotides harboring phenotype-associated variants.

### 3.3. Regulatory Function of ncRNAs Aligning with the TRIM33 Gene

In 28 cases, sequence similarity between the *TRIM33* gene and ncRNAs was found in ncRNA gene regulatory elements. Specifically, the hits involved 12 lncRNA gene enhancers, 1 lncRNA gene enhancer + CCCTC-binding factor (CTCF), 1 lncRNA gene transcription factor binding site, 13 lncRNA gene promoters and 1 lncRNA gene CTCF (Table 2).

It follows that such sequence complementarity could potentially lead to an interaction between *TRIM33* transcripts and these ncRNA gene regulatory regions in the nucleus, disrupting the organization of chromatin architecture and preventing the binding of transcription factors and other key mediators that are critically involved in enhancer-promoter communication [29]. All of these events can prevent the recruitment of RNA polymerase II to the promoter site of ncRNA genes and thus impair their expression.

**Figure 2:**
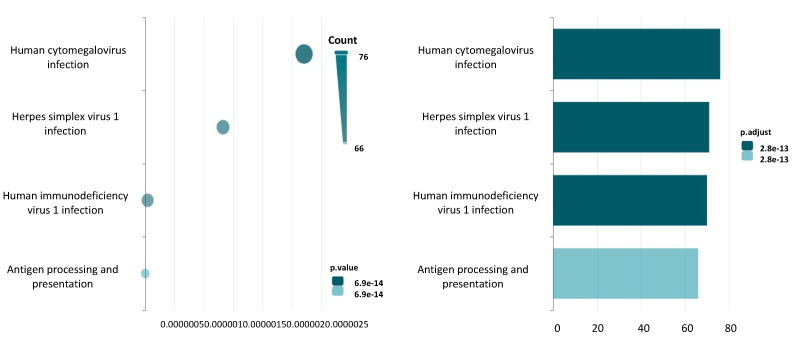
Enrichment analysis pathways associated with lncRNAs being complementary to *TRIM33* in a gene regulatory region. The figure was created with NcPath.

To determine which pathways may be potentially disrupted by *TRIM33* sequence alignment, KEGG analysis was run by using NcPath for the detected lncRNAs harboring hits on their gene regulatory regions. The pathways involved regarded antigen processing and presentation (*p* value = 6.89419105768181e-14), human immunodeficiency virus 1 infection (*p* value = 2.0404015074785747e-08), herpes simplex virus 1 infection (*p* value = 9.94404432054867e-07), and human cytomegalovirus infection (*p* value = 2.7087345449239465e-06) (Figure 2).

### 3.4. QmRLFS-Finder Analysis

QmRLFS-finder is a free web program that calculates the prediction of R-loop formation in a nucleic acid sequence of interest [27]. R-loops are transient three-stranded RNA–DNA hybrids that form during DNA replication, transcription and repair and are involved in genome stability and the control of gene transcription [30]. Due to their importance in processes such as transcription-coupled and RNA template-based DNA break repair, the imbalance in R-loop formation has been studied in cancer, neurodegenerative diseases and autoimmune diseases. Both too many and too few R-loops can destabilize chromatin integrity and thus increase the risk of tumorigenesis [31]. R-loops usually form near transcription initiation sites (promoters), but also at transcription termination sites, telomeric and centromeric regions and in mitochondrial DNA [31].

To determine whether the *TRIM33* nucleotide sequence could match an ncRNA nucleotide sequence predicted to form R-loops, QmRLFS finder analysis was performed, selectively focusing on matches in regulatory sequences of ncRNA genes. It is noteworthy that the results did not identify an R-loop-forming site in the query sequence (Table 2), which does not support a role for *TRIM33* transcripts in disrupting this mechanism.

### 3.5. Spliceator Analysis

To test whether the alignment of the ncRNA nucleotide sequences to different sites of intron 1 of the *TRIM33* gene could interfere with splicing, Spliceator analysis was performed.

Spliceator is a freely available computational tool that allows the prediction of 5′ donor splice sites and 3′ acceptor splice sites in a sequence of interest [28]. This system uses a convolutional neural network with an estimated accuracy of 89–92%. Briefly, the nucleotide sequences of *TRIM33* genes that matched ncRNAs from the Ensembl.org database were identified. As mentioned above, all of them were located in intron 1 and spanned the regions from 9987 to 10,297 bp, from 23,274 to 23,253 bp and from 30,709 to 31,395 bp. For each of the 3 nucleotide sequences, a separate analysis was performed with Spliceator, selecting the following parameters: reliability donor 98 %; acceptor 98 %; model: 200; splice site: both.

Interestingly, 9 acceptor splice sites were predicted on intron 1 of the *TRIM33* gene with a score between 0.982 and 0.992 (Figure S1). Therefore, binding of ncRNAs at such sites could potentially disrupt the mechanism of splicing and lead to intron retention or generate alternatively spliced isoforms.

### 3.6. MiR-142-3p as a Multiple Interactor with TRIM33 Gene and TRIM33 Gene-Aligned lncRNAs Dysregulated in Dermatomyositis

LncRNAs and other smaller RNAs, which include microRNAs (miRNAs), can be involved in a network of competition for shared nucleotide sequences according to the competitive endogenous RNA (ceRNA) hypothesis. Briefly, miRNAs mainly act as post-transcriptional regulators of gene expression by binding to specific miRNA responsive elements (MREs) located in the 3′-UTR regions of mRNA [32]. MicroRNAs can in turn be siphoned off by other ncRNAs, including transcribed pseudogenes and lncRNAs that share identical MREs. An imbalance in this network can lead to altered mRNA translation and has been linked to carcinogenesis and autoimmunity [33]. 

To determine whether altered expression of *TRIM33* could contribute to the aberrant transcriptomic profile found in experiments on DM, RNAInter analysis was performed to detect RNA–RNA interactions. The *TRIM33* mRNA transcript and the *TRIM33* gene-targeting lncRNAs, which are also dysregulated in DM according to a recent bioinformatic study [34], were used as inputs. By searching for RNAs interacting with *TRIM33* mRNA, a total of 735 interactions were found (Table S2).

These results were then compared with those from previously published experimental studies on plasma or muscle samples from patients with DM or other IIMs [22,35,36,37,38,39]. As shown in Table 3, a total of 28 correlations were found.

Interestingly, the genes *TRIM33*, *NNT-AS1* and *PAXBP1-AS1* were all predicted to interact with the miR-142-3p, while the lncRNA gene *MKLN1-AS* was predicted to interact with metazoan signal recognition particle (SRP) RNA. The SRP is a well-known antigen in immune-mediated necrotizing myopathy and anti-SRP antibodies can be detected in a minority of IIM patients with cardiac involvement and no cancer association [40].

## 4. Discussion

The results of this pivotal in silico study indicate that the expression of TIF1γ in DM patients may depend on a complex network of DNA–RNA and RNA–RNA interactions. This scenario could occur early in cells prone to malignant transformation and trigger a primitive antitumor response. Indeed, TIF1γ appears to act as a tumor suppressor by antagonizing the transforming growth factor (TGF)-β/Smad pathway and the Wingless-INT (Wnt)/β-catenin pathway [10,12]. Specifically, TIF1γ can monoubiquitinate Smad4 and prevent the formation of the Smad2/3/4 complex in the nucleus, thereby antagonizing the action of TGF-β [11,12]. Moreover, TIF1γ can contribute to β-catenin proteasome degradation and turn off Wnt signaling in cancer cells. Consistent with its tumor suppressive function, TIF1γ expression is significantly reduced in most tumors, including lung cancer, pancreatic cancer, liver cancer, and glioblastoma, while it is increased in other tumors such as colorectal cancer and breast cancer [10,12]. In addition, some studies suggest that dysregulation of TIF1γ may be an early event in tumorigenesis that contributes significantly to cancer progression [41,42,43]. These results can be explained by the dual role of TIF1γ in tumor growth and immune surveillance. TIF1γ regulates the proliferation and differentiation of hematopoietic cells and can be considered as a promoter of B-lymphoblastic leukemia [13]. In the presence of interleukin-6 (IL-6), TIF1γ can redirect T lymphocyte differentiation toward T helper 17 (Th17) subtypes by antagonizing TGF-β signaling to the Smad2/3/4 complex, which is critical for T regulatory (Treg) differentiation [16]. The imbalance of Th17/Treg subsets may eventually trigger the development of autoimmunity. Moreover, TIF1γ may control the activation of macrophages and invariant natural killer T cells (iNKT), which are involved in immune surveillance against tumors and in the development of autoimmune diseases [12]. Thus, TIF1γ overproduction may be an attempt to curb tumor development by impeding crucial anabolic pathways in cancer cells and triggering a robust immune response. In a proportion of cancer patients, tolerance to TIF1γ may be disrupted, with the subsequent appearance of anti-TIF1γ antibodies in serum, which may cross-react with TIF1γ synthesized in skin or skeletal muscle [44]. Anti-TIF1γ antibodies can be detected in up to 35% of adult DM patients who are frequently diagnosed with lung, uterine, colon, breast, ovarian, and lymphoma cancers within three years [2,10,45]. Exposure to ultraviolet light or other cellular stressors together with a favorable genetic and immunological background and an attempt at myofiber differentiation and regeneration may increase the production of the protein in skin and skeletal muscle [44,46,47,48]. However, evidence for the tissue expression of TIF1γ in cancer-associated DM is limited and conflicting. Although the results of an immunohistochemical study did not reveal a significant correlation between muscular expression of TIF1γ and cancer risk in DM [44], other data suggested an association between somatic mutations of the *TRIM33* gene and paraneoplastic DM [14,15].

In this analysis, the *TRIM33* gene appears to have sequence complementarity with human ncRNA genes, mainly lncRNAs, some of which play a critical role in tumor development and progression. LncRNAs consist of a class of RNA transcripts longer than 200 nucleotides that are generally not translated into proteins. Given their diverse roles in processes related to cell development and differentiation, lncRNAs have been extensively studied in cancer. Indeed, lncRNAs can hybridize with miRNAs, mRNAs, DNA and proteins and function as regulators of gene expression, mRNA stability, alternative splicing and translation, as well as protein scaffolds or decoys [33]. In cancer studies, deregulation of lncRNAs has been reported to be related to cell transformation and the acquisition of a stem cell phenotype [49].

On the other hand, lncRNA alterations have been documented in autoimmune diseases such as rheumatoid arthritis, systemic lupus erythematosus, psoriasis and type 1 diabetes mellitus [50]. In this context, lncRNAs may play a pathogenic role by controlling the expression of immune-related genes, such as those encoding cytokines, in response to stimulation by Toll-like receptors (TLRs) and consequently regulating the differentiation and activation of immune cells.

A few studies have examined the transcriptomic changes that occur in DM [21,22,34,51]. In a recent paper, Huang et al. reported the possible dysregulation of 3835 lncRNAs and 52 miRNAs in DM according to the ceRNA theory [34]. Interestingly, four of the lncRNAs reported by the authors were also associated with the *TRIM33* gene after this analysis. These included the lncRNA *NNT-AS1*, which is upregulated in several cancers and associated with poor overall survival [52], the lncRNA *MKLN1-AS*, which is overexpressed in hepatocellular carcinoma with poor prognosis [53], the lncRNA *LINC01206*, whose expression is increased in samples of squamous cell carcinoma of the lung [54], and *PAXBP1-AS1*, whose dysregulation has been observed in triple-negative breast cancer [55]. Thus, the increase in key genes in DM may be related to lncRNAs, some of which are complementary to the nucleotide sequence of *TRIM3*3. The major limitation in this study is the lack of characterization of the ncRNA profile of patients with paraneoplastic DM. Conversely, no matches can be found between *TRIM33*-aligning lncRNAs and lncRNAs dysregulated with an absolute fold-change > 5 according to a microarray analysis of DM muscle samples by Peng et al. [21]. It is plausible that the overexpression of *TRIM33* could be an attempt to curb the production of lncRNAs associated with tumor progression. Interestingly, this analysis shows that *TRIM33* may align to the lncRNA genes *MKLN1-AS* and *PAXBP1-AS1* in the enhancer and promoter regions, respectively, which could prevent further expression of these genes in malignant cells.

On the other hand, the complementation of lncRNAs with *TRIM33* mRNA transcripts could prevent their translation and further accelerate carcinogenesis. Indeed, the Cancer Genome Atlas (TCGA) dataset (https://www.cancer.gov/ccg/research/genome-sequencing/tcga accessed on 6 June 2024) shows reduced expression of *TRIM33* in various tumor types due to the loss of expression variants. These data are consistent with the loss of heterozygosity (LOH) in the *TRIM33* gene reported by Pinal-Fernandez et al. in patients with cancer-associated myositis [15]. These events could reduce the expression of the mutant isoforms of TIF1γ—and thus reduce the immunogenicity of the malignant cell—and upregulate the Wnt/β-catenin TGF-β/Smad signaling pathways that promote cancer cell survival.

This analysis also shows that nucleotide alignment between *TRIM33* and ncRNA genes occurs exclusively at intronic sites and may involve nine acceptor splice sites. Although this result can be explained based on the selected FASTA sequence of the *TRIM33* gene, which was restricted to the coding portion, complementation of multiple lncRNAs at *TRIM33* intron 1 may reflect interference with canonical and alternative splicing, perhaps reducing the expression of functional mRNAs or generating alternatively spliced isoforms of TIF1γ that would promote autoimmunity. This hypothesis is consistent with data from other next-generation sequencing and whole-exome sequencing experiments in cancer patients, which show that the formation of TIF1γ neoantigens due to somatic mutations of the *TRIM33* gene could trigger the production of anti-TIF1γ antibodies and form the basis for DM [14,15]. Specifically, the paper by Cordel et al. reports the occurrence of two variants at the donor and acceptor sites of *TRIM33* intron 18 in two solid cancer samples, which may impair canonical splicing and generate new protein isoforms with immunogenic epitopes [14]. However, the intronic sites of the *TRIM33* gene predicted to bind to ncRNAs by this computational analysis do not include intron 18. Indeed, some lncRNAs have been observed to act as natural antisense transcripts and interact with cis-acting elements in precursor mRNAs (pre-mRNAs) through base pair complementation, thus affecting the recruitment of splicing factors or the selection of the splice sites [56]. Additionally, there is evidence that pre-mRNAs of autoantigens often undergo alternative splicing phenomena and that such modifications may enhance the immunogenicity of self-proteins and influence antigen recognition and binding by dendritic cells [57].

To date, there are no clear associations between the expression of a specific splice variant of the *TRIM33* gene and the risk of cancer or DM. The Ensembl.org database reports six alternative spliced variants of the *TRIM33* gene (https://www.ensembl.org/Homo_sapiens/Gene/Summary?db=core;g=ENSG00000197323;r=1:114392790-114511203;t=ENST00000358465 accessed on 19 May 2024), of which only transcripts ENST00000358465, ENST00000369543 and ENST00000448034 encode a protein of 1127aa, 1110aa and 888aa, respectively, while the other transcripts have an undefined protein coding sequence. Consultation of the Biociphers tool provided by the UCSB Genome Browser reveals that transcripts ENST00000448034, ENST00000476908, ENST00000478032 and ENST00000492227 originate from exon 4 upwards and thus skip exon 1 and 2, which could be affected by defective splicing on intron 1 (Figure S2).

Binding of ncRNAs to *TRIM33* intron 1 could indeed trigger intron retention, which has been described in the context of cancer and other diseases because it alters transcriptome plasticity and the expression of oncosuppressor genes [58]. However, evidence for the mechanism of intron retention of the *TRIM33* gene as pathogenic in cancer and DM is lacking.

*TRIM33* transcripts could complement with gene regulatory regions of lncRNA genes that, according to NcPath analysis, preside over signaling pathways involved in antigen presentation and antiviral response. DM is known to be associated with a type I interferon signature, which in turn is related to the overactivity of plasmacytoid dendritic cells (pDCs) in skeletal muscle or skin [59]. In addition to producing type I interferons, pDCs can also function as antigen-presenting cells and activators of T effector or regulatory cells and therefore play a central role in autoimmunity and cancer [60].

Finally, this study revealed that 28 miRNAs dysregulated in plasma or muscle biopsy samples from patients with IIMs [22,35,37,38,39] are predicted to bind to *TRIM33*. Among them, miRNA-142-3p was found hypoexpressed in plasma exosomes from IIM patients compared to healthy donors [35]. According to RNAInter analysis, miR-142-3p could complement with either TRIM33 mRNA or the lncRNAs NNT-AS1 and PAXBP1-AS1, which appear to be overexpressed in malignant cells and DM [34,52,55]. The overproduction of these lncRNAs, which compete with miRNA-142-3p, could eventually lead to increased TIF1γ translation and activation of autoimmune pathways.

MiR-142 is an important regulator of the cell cycle and is also involved in hematopoietic cell function. Specifically, miR-142-3p interferes with myeloid differentiation, neutrophil and mast cell functions, and T and B cell homeostasis. In the tumor microenvironment, miR-142-3p may antagonize the function of tumor-associated macrophages (TAMs) by suppressing the TGF-β axis, thereby impairing the tumor-supportive role of these cells, whereas the miR-142-5p isoform appears to have antagonistic effects [61,62]. On the other hand, a study in transgenic mice with severe myositis reported the increased expression of miR-142-3p, which was associated with the upregulation of nuclear factor-kB (NF-κB)-regulated genes and the downregulation of dystrophin [63].

Both ncRNAs and protein products can be transported between cancer cells, immune cells, and other tissues, such as skeletal muscle, via exosomes [61]. Recently, researchers have attempted to characterize the profile of exosomal RNA in the plasma of patients with DM, some of whom were positive for anti-TIF1γ antibodies [35,36,64]. Indeed, the results of these studies showed the altered expression of lncRNAs and miRNAs. Of note, two dysregulated miRNAs characterized in the plasma exosomes of cancer-associated myositis (hsa-let-7f-5p and hsa-miR-143-3p) were also among the predicted interactors of *TRIM33* mRNA according to this analysis. Conversely, the top differentially expressed lncRNAs reported by these studies were not found among the *TRIM33*-aligning lncRNAs. It is unclear whether the selected population (DM patients without cancer) may have contributed to the conflicting results.

**Figure 3:**
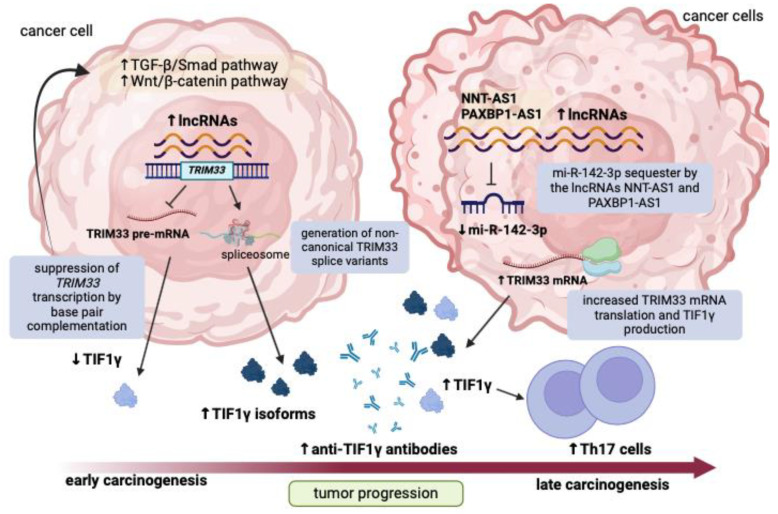
Interactome network potentially leading to cancer progression and concomitant autoimmune phenomena in paraneoplastic DM. In the early phase of carcinogenesis, malignant cells may exhibit an altered transcriptome characterized by the overexpression of a number of lncRNAs. Some of these lncRNAs may have nucleotide sequence complementarity with the *TRIM33* gene and affect its transcription and alternative splicing by binding to *TRIM33* intronic sites. The subsequent reduction in the production of functional isoforms of TIF1γ may accelerate cancer progression by upregulating the transforming growth factor-β/Smad pathway and the Wingless-INT/β-catenin pathway in tumor cells and generate TIF1γ neoepitopes that could activate cancer-surrounding immune cells. In addition, lncRNA transcripts that migrate into the cytosol of cancer cells can establish an RNA–RNA network by sequestering miRNAs known to regulate TIF1γ translation. Specifically, the overexpression of lncRNA *NNT-AS1* and *PAXBP1-AS1* may prevent mi-R-142-3p from sponging *TRIM33* transcripts. This event may eventually lead to the promotion of the translation of alternative *TRIM33* splice variants and the production of TIF1γ self-epitopes, which together with the imbalance of T cell subpopulations can trigger a cross-reactive immune response in skeletal muscle and skin. Abbreviations: C: cytosol; lncRNAs: long noncoding RNAs; mRNA: messenger RNA; miRNA: micro-RNA; N: nucleus; pre-mRNA: precursor messenger RNA; TGF-β: transforming growth factor-β; TIF1γ: transcription intermediary factor 1 gamma; Th17: T helper 17; *TRIM33*: tripartite motif containing 33; Wnt: Wingless-INT. The figure was created with BioRender.com accessed on 8 June 2024.

**Figure 4:**
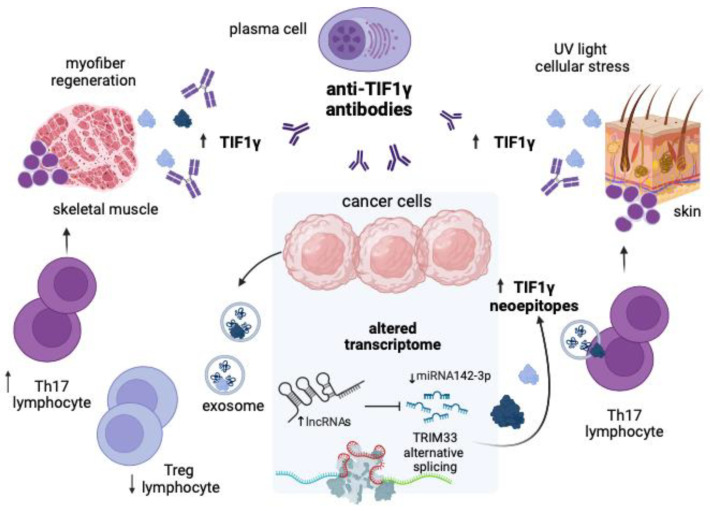
Possible mechanism to explain the production of anti-TIF1γ antibodies in cancer patients with DM. The main linchpin of the hypothesis supported by the data of this study lies in the altered transcriptomic profile of cancer cells, characterized by an unbalanced expression of lncRNAs that have the potential to target directly the *TRIM33* gene or miRNAs involved in its regulation. The *TRIM33* nucleotide sequence could complement with 100 ncRNAs in intronic regions and undergo defective alternative splicing with the potential generation of neoepitopes. As a result, the expression of canonical and noncanonical isoforms of TIF1γ could eventually be increased. Transcripts and produced proteins, including TIF1γ, can be transported between cancer cells and immune cells in exosomes and influence the phenotype of these cells. Specifically, T lymphocytes can be induced to differentiate into Th17 cells to the detriment of Treg cells, which helps plasma cells produce autoantibodies. This disruption of immune tolerance along with the overexpression of TIF1γ in skeletal muscle and skin after exposure to stressors may be the basis for cross-reactivity phenomena and the appearance of DM. Abbreviations: lncRNA: long noncoding RNA; miRNA: micro-RNA; Th17: T helper 17; Treg: T regulatory; TIF1γ: transcription intermediary factor 1 gamma; UV: ultraviolet. The figure was created with BioRender.com accessed on 8 June 2024.

To resume, this research paves the way for a new hypothesis that may explain the occurrence of DM as a paraneoplastic phenomenon (Figure 3 and Figure 4).

Cancer cells in the very early phase of malignant transformation may express a variety of lncRNA transcripts, some of which overlap with the nucleotide sequence of *TRIM33*. In the nucleus, this event may initially repress the transcription of the gene, which has a tumor-suppressive effect, by direct base pair complementation and can accelerate cancer development. Binding of *TRIM33* to intronic sites could disrupt the alternative splicing mechanism and contribute to the generation of dysfunctional isoforms of TIF1γ, which may also behave as neoantigens. Once dysregulated lncRNA transcripts enter the cytosol, they may sequester miRNAs that normally bind *TRIM33* mRNA and increase its expression. However, it may also be possible that sequence complementarity between lncRNAs and *TRIM33* transcripts prevents the latter from being translated into final proteins by a competing mechanism. Such transcriptomic alterations may be exchanged between malignant cells and immune cells via exosomes. After endocytosis of cancer-related exosomes containing abnormally expressed lncRNA and miRNA transcripts, immune cells surrounding the tumor may be phenotypically altered to become autoreactive.

However, the present analysis has several limitations. The first arises from the in silico nature of the study, the results of which should be confirmed by in vitro or ex vivo experiments such as Western blot or Chip-Seq analyses. In addition, the study primarily aimed to investigate direct DNA–RNA and RNA–RNA base pair complementation. Possible interactions with RNA-binding proteins or other intermediates that might come into contact with specific RNA conformational domains [65] were not investigated. Furthermore, the detection of anti-TIF1γ antibodies in adult but not adolescent patients with cancer-associated DM is unclear, and the underlying mechanisms should be better elucidated. Another limitation is the lack of characterization of the contribution of the gene *TRIM24* encoding TIF1α, which can also induce dual immunoreactivity [11]. Finally, the analysis was restricted to the coding FASTA sequence of the *TRIM33* gene and the complementation of ncRNAs on flanking 3′UTRs and 5′UTRs known for regulatory activity was not examined. All these points represent important issues that need to be considered in the future research agenda.

## 5. Conclusions

In conclusion, the results of this in silico analysis show sequence complementarity and predict interactions between the *TRIM33* gene and human ncRNAs dysregulated in cancer and DM. These data suggest a very complex “nucleic acid battle” in which *TRIM33* transcripts may complement with and prevent transcription of several lncRNA genes overexpressed in cancer, which in turn may restrict *TRIM33* expression directly or by sequestering *TRIM33* mRNA-binding microRNAs, or lead to *TRIM33*-defective splicing mechanisms. The sum of these events can lead to the emergence of *TRIM33* isoforms, to an imbalance in the T cell subpopulation and to the acceleration of cancer development or the occurrence of autoimmune phenomena. Indeed, such an altered transcriptome profile could be due to a primitive attempt to counteract tumor development and eventually lead to the formation of autoreactive immune cells that cross-react against skeletal muscle and skin autoantigens.

Thus, the overproduction of anti-TIF1γ antibodies in cancer patients who develop DM may be explained by dysregulated cross-talk between malignant and immune cells. In this context, the search for transcripts in circulating exosomes could provide scientists with potential biomarkers to monitor or even predict disease progression. In addition, the results of this study may give some clues to the therapeutic potential of exosomes, which could be used as vectors for desired coding or noncoding transcripts to normalize epigenetic and biomolecular pathways in cancer-associated DM.

## Figures and Tables

**Table 1:** Noncoding RNA genes aligning with *TRIM33* nucleotide sequence having known associations with human disease according to the GeneCards database. If the matched transcripts are more than 1, length and % ID are given as mean ± standard deviation.

Gene	Type	Tissue Expression	Subcellular Localization	Associated Disease	No. of Matched Transcripts	Alignment Length (bp)	%ID	Hit on a Phenotype-Associated Variant
***NNT-AS1***	lncRNA	almost ubiquitous	nucleus, cytoskeleton, mitochondria, extracellular, plasma membrane	cervical cancer, ovarian cancer, osteogenic sarcoma, hepatocellular cancer, colorectal cancer	1	676	91.8	no
***HELLPAR***	lncRNA	skeletal muscle tissue, liver	nucleus	HELLP syndrome	3	403 ± 170.6	93.9 ± 0.5	no
***GTF3C2-AS2***	lncRNA	almost ubiquitous	/	leukoencephalopathy with vanishing white matter; retinitis pigmentosa	1	671	92.1	no
***LINC01551***	lncRNA	overexpressed in the brain	/	hypertropia	4	308.5 ± 0.5	96.7 ± 3.3	no
***LINC01116***	lncRNA	almost ubiquitous (overexpressed in the kidney)	nucleus, cytoskeleton, extracellular	breast cancer, glioblastoma, glioma, lung cancer, ovarian squamous cell carcinoma	2	310.5 ± 2.1	95.8 ± 4.1	no
***LINC00824***	lncRNA	overexpressed in whole blood, intestine and spleen	/	primary spontaneous pneumothorax	2	297 ± 0	98.9 ± 0	no
***PCBP1-AS1***	lncRNA	almost ubiquitous	nucleus, extracellular	retinitis pigmentosa	1	279	96.7	no
***MIRLET7BHG***	lncRNA	almost ubiquitous	nucleus, extracellular	brachydactyly type b2	1	304	94.7	no
***LINC01285***	lncRNA	almost ubiquitous	/	monkeypox virus infection	1	305	94.7	no
***SILC1***	lncRNA	overexpressed in the brain	nucleus	gastric cancer	1	267	97	no

Abbreviations: bp: base pairs; HELLP: hemolysis, elevated liver function tests, and low platelets; lncRNA: long noncodingRNA; % ID: percentage of identity.

**Table 2:** Noncoding RNA genes aligning with *TRIM33* nucleotide sequence in a gene regulatory region according to the Ensembl.org database.

Gene	Transcript	Type	Tissue Expression	Localization	Type of Regulatory Region	R-Loops
*ZNF584-DT*	ENST00000593393.1	lncRNA	testis, endometrium	/	enhancer, CTCF	0
*MKLN1-AS*	ENST00000416220.7	lncRNA	almost ubiquitous	nucleus	enhancer	0
*ENSG00000254258*	ENST00000522373.1	lncRNA	almost ubiquitous	/	enhancer	0
*ARHGEF35-AS1*	ENST00000650250.1	lncRNA	almost ubiquitous	/	enhancer	0
*ENSG00000284294*	ENST00000638287.1	lncRNA	sural nerve, colonic epithelium, bone marrow cell	/	enhancer	0
*ENSG00000284294*	ENST00000640084.1	lncRNA	sural nerve, colonic epithelium, bone marrow cell	/	enhancer	0
*ENSG00000287839*	ENST00000668003.2	lncRNA	whole blood, intestine	/	enhancer	0
*ENSG00000285658*	ENST00000648317.1	lncRNA	bone marrow cell, skeletal muscle tissue, colonic epithelium	/	TFB	0
*OR2A1-AS1*	ENST00000468575.1	lncRNA	almost ubiquitous	/	enhancer	0
*MRPL20-DT*	ENST00000607307.1	lncRNA	almost ubiquitous	/	promoter	0
*ANXA2R-OT1*	ENST00000702244.1	lncRNA	almost ubiquitous	cytosol	enhancer	0
*ENSG00000215014*	ENST00000366221.3	lncRNA	overexpressed in testis	/	promoter	0
*XACT*	ENST00000674361.1	lncRNA	testis, colon, nervous system	nucleolus	CTCF	0
*ENSG00000267353*	ENST00000588717.1	lncRNA	myometrium, endometrium	/	promoter	0
*LINC00501*	ENST00000425388.1	lncRNA	almost ubiquitous	extracellular	promoter	0
*ARIH2OS*	ENST00000647812.1	lncRNA	testis, ovary	nucleus, plasma membrane, extracellular	promoter	0
*ENSG00000287277*	ENST00000664549.1	lncRNA	colonic epithelium, bone marrow cell, sural nerve	/	enhancer	0
*ENSG00000287277*	ENST00000669799.1	lncRNA	colonic epithelium, bone marrow cell, sural nerve	/	enhancer	0
*ENSG00000287277*	ENST00000666057.1	lncRNA	colonic epithelium, bone marrow cell, sural nerve	/	enhancer	0
*ENSG00000287277*	ENST00000665216.1	lncRNA	colonic epithelium, bone marrow cell, sural nerve	/	enhancer	0
*PAXBP1-AS1*	ENST00000691027.1	lncRNA	almost ubiquitous	/	promoter	0
*PAXBP1-AS1*	ENST00000653345.1	lncRNA	almost ubiquitous	/	promoter	0
*PAXBP1-AS1*	ENST00000660366.1	lncRNA	almost ubiquitous	/	promoter	0
*PAXBP1-AS1*	ENST00000440052.5	lncRNA	almost ubiquitous	/	promoter	0
*PAXBP1-AS1*	ENST00000458479.1	lncRNA	almost ubiquitous	/	promoter	0
*PAXBP1-AS1*	ENST00000665654.1	lncRNA	almost ubiquitous	/	promoter	0
*PAXBP1-AS1*	ENST00000662524.1	lncRNA	almost ubiquitous	/	promoter	0
*PAXBP1-AS1*	ENST00000665120.1	lncRNA	almost ubiquitous	/	promoter	0

Abbreviations: CTCF: CCCTC-binding factor; lncRNA: long noncoding RNA; TFB: transcription factor binding site.

**Table 3:** miRNAs predicted to interact with *TRIM33* in RNAInter analysis and shown to be dysregulated in IIMs in previous experimental studies. The results are sorted by RNAInter score.

miRNA	RNAInter Score	Results from Other Studies	Methods	Reference
hsa-miR-142-3p	0.4564	downregulated in plasma exosomes of patients with IIMs compared to controls	NGS	[35]
hsa-miR-3613-5p	0.4528	downregulated in plasma exosomes of patients with IIMs compared to controls	NGS	[35]
hsa-miR-196a-5p	0.4457	downregulated in PM muscle biopsy samples but upregulated in HSkMM after exposure to 20% PM serum	miRNAome analysis	[22]
hsa-miR-21-5p	0.4212	upregulated in DM and ASyS muscle biopsy samples compared to controls	NanoString nCounter system	[38]
hsa-miR-30e-5p	0.4139	upregulated in HSkMM after stimulation with 20% PM serum	miRNAome analysis	[22]
hsa-miR-32-5p	0.4086	upregulated in plasma exosomes of patients with IIMs compared to controls	NGS	[35]
hsa-miR-491-5p	0.4086	upregulated in HSkMM after stimulation with 20% PM serum	miRNAome analysis	[22]
hsa-let-7f-5p	0.4017	upregulated in plasma exosomes of CAM patients compared to non-CAM IIM patients	NGS	[35]
hsa-let-7a-5p	0.4017	downregulated in plasma exosomes of patients with IIMs compared to controls	NGS	[35]
hsa-miR-133b	0.3649	upregulated and downregulated in IIM muscle biopsy samples compared to controls according to two different studies	NanoString nCounter system;miRNA microarray and qPCR	[38,39]
hsa-miR-30d-5p	0.3649	downregulated in PM muscle biopsy samples but upregulated in HSkMM after exposure to 20% PM serum	miRNAome analysis	[22]
hsa-miR-30a-5p	0.3649	downregulated in PM muscle biopsy samples but upregulated in HSkMM after exposure to 20% PM serum	miRNAome analysis	[22]
hsa-miR-133a-3p	0.3649	downregulated in IIM muscle biopsy samples comparedto controls	miRNA microarray and qPCR	[39]
hsa-miR-30e-3p	0.3579	downregulated in muscle biopsy samples from DM patients and correlated with the IFN-I signature	NanoString nCounter system	[38]
hsa-miR-30a-3p	0.3579	downregulated in muscle biopsy samples from DM patients and correlated with the IFN-I signature	NanoString nCounter system	[38]
hsa-miR-498-5p	0.3097	upregulated in plasma samples from PM/DM patients after immunosuppressive treatment	miRNA microarrayand qRT-PCR	[37]
hsa-miR-495-3p	0.2986	upregulated in IBM muscle biopsy samples compared to DMand controls	NanoString nCounter system	[38]
hsa-miR-130a-3p	0.2129	upregulated in HSkMM after stimulation with 20% PM serum	miRNAome analysis	[22]
hsa-miR-543	0.2129	upregulated in IBM muscle biopsy samples compared to DMand controls	NanoString nCounter system	[38]
hsa-miR-143-3p	0.2129	downregulated in plasma exosomes of CAM patients compared to non-CAM patients	NGS	[35]
hsa-miR-302d-3p	0.2129	downregulated in muscle biopsy samples from IIM patients compared to controls	NanoString nCounter system	[38]
hsa-miR-382-5p	0.2129	upregulated in IIM muscle biopsy samples compared to controls	NanoString nCounter system	[38]
hsa-miR-411-5p	0.2129	upregulated in IBM muscle biopsy samples compared to DMand controls	NanoString nCounter system	[38]
hsa-miR-27a-3p	0.1862	downregulated in plasma exosomes of DM patients compared to PM/ASyS patients	NGS	[35]
hsa-miR-26a-5p	0.1862	upregulated in HSkMM after stimulation with 20% PM serum	miRNAome analysis	[22]
hsa-miR-320e	0.1619	uregulated in DM muscle biopsy samples compared to controls	NanoString nCounter system	[38]
hsa-miR-361-3p	1E-10	upregulated in DM muscle biopsy samples and associated with IFN-I signature	NanoString nCounter system	[38]
hsa-miR-221-3p	1E-10	upregulated in HSkMM after stimulation with 20% PM serum; differentially expressed in muscle biopsy samples of IIM patients compared to controls (mainly upregulated)	miRNAome analysis NanoString nCounter system	[22,38]

Abbreviations: ASyS: antisynthetase syndrome; CAM: cancer-associated myositis; DM: dermatomyositis; HSkMM: human skeletal muscle myoblasts; IBM: inclusion body myositis; IFN I: type I interferon; IIMs: idiopathic inflammatory myopathies; miR: microRNA; NGS: Next Generation Sequencing; PM: polymyositis; qPCR: quantitative polymerase chain reaction; qRT-PCR: quantitative real-time polymerase chain reaction.

## Data Availability

Data is contained within the article and Supplementary Materials.

## Supplementary Materials

The following supporting information can be downloaded at: https://www.mdpi.com/article/10.3390/jpm14060628/s1, Table S1: List and main features of human ncRNAs aligning to *TRIM33* nucleotide sequence; Table S2: List of RNA interactors with *TRIM33* mRNA transcript according to RNAInter analysis and interaction network (top 100 interactions) of *TRIM33* mRNA and miR-142-3p. Figure S1: Splice sites on *TRIM33* nucleotide sequences matching ncRNAs identified by Spliceator analysis. Figure S2: Splice graph of *TRIM33* transcripts according to Biociphers analysis.

## References

[1] Lundberg I.E., Tjärnlund A., Bottai M., Werth V.P., Pilkington C., de Visser M., Alfredsson L., Amato A.A., Barohn R.J., Liang M.H. (2017). 2017 European League Against Rheumatism/American College of Rheumatology Classification Criteria for Adult and Juvenile Idiopathic Inflammatory Myopathies and Their Major Subgroups. Arthritis Rheumatol..

[2] Patasova K., Lundberg I.E., Holmqvist M. (2023). Genetic Influences in Cancer-Associated Myositis. Arthritis Rheumatol..

[3] Dourado E., Bottazzi F., Cardelli C., Conticini E., Schmidt J., Cavagna L., Barsotti S. (2023). Idiopathic Inflammatory Myopathies: One Year in Review 2022. Clin. Exp. Rheumatol..

[4] Carstens P.O., Schmidt J. (2014). Diagnosis, Pathogenesis and Treatment of Myositis: Recent Advances. Clin. Exp. Immunol..

[5] Malik A., Hayat G., Kalia J.S., Guzman M.A. (2016). Idiopathic Inflammatory Myopathies: Clinical Approach and Management. Front. Neurol..

[6] Ernste F.C., Reed A.M. (2013). Idiopathic Inflammatory Myopathies: Current Trends in Pathogenesis, Clinical Features, and Up-to-Date Treatment Recommendations. Mayo Clin. Proc..

[7] Dalakas M.C. (2015). Inflammatory Muscle Diseases. N. Engl. J. Med..

[8] Fiorentino D.F., Chung L.S., Christopher-Stine L., Zaba L., Li S., Mammen A.L., Rosen A., Casciola-Rosen L. (2013). Most Patients with Cancer-Associated Dermatomyositis Have Antibodies to Nuclear Matrix Protein NXP-2 or Transcription Intermediary Factor 1γ. Arthritis Rheum..

[9] Fiorentino D.F., Kuo K., Chung L., Zaba L., Li S., Casciola-Rosen L. (2015). Distinctive Cutaneous and Systemic Features Associated with Antitranscriptional Intermediary Factor-1γ Antibodies in Adults with Dermatomyositis. J. Am. Acad. Dermatol..

[10] Kotobuki Y., Tonomura K., Fujimoto M. (2020). Transcriptional Intermediary Factor 1 (TIF1) and Anti-TIF1γ Antibody-Positive Dermatomyositis. Immunol. Med..

[11] De Vooght J., Vulsteke J.B., De Haes P., Bossuyt X., Lories R., De Langhe E. (2020). Anti-TIF1-Γautoantibodies: Warning Lights of a Tumour Autoantigen. Rheumatology.

[12] Yu C., Ding Z., Liang H., Zhang B., Chen X. (2019). The Roles of TIF1γ in Cancer. Front. Oncol..

[13] Kusy S., Gault N., Ferri F., Lewandowski D., Barroca V., Jaracz-Ros A., Losson R., Romeo P.H. (2011). Adult Hematopoiesis Is Regulated by TIF1γ, a Repressor of TAL1 and PU.1 Transcriptional Activity. Cell Stem Cell.

[14] Cordel N., Derambure C., Coutant S., Mariette X., Jullien D., Debarbieux S., Chosidow O., Meyer A., Bessis D., Joly P. (2021). TRIM33 Gene Somatic Mutations Identified by next Generation Sequencing in Neoplasms of Patients with Anti-TIF1γpositive Cancer-Associated Dermatomyositis. Rheumatology.

[15] Pinal-Fernandez I., Ferrer-Fabregas B., Trallero-Araguas E., Balada E., Martínez M.A., Milisenda J.C., Aparicio-Español G., Labrador-Horrillo M., Garcia-Patos V., Grau-Junyent J.M. (2018). Tumour TIF1 Mutations and Loss of Heterozygosity Related to Cancer-Associated Myositis. Rheumatology.

[16] Jiang Y., Wang X., Dong C. (2019). Molecular Mechanisms of T Helper 17 Cell Differentiation: Emerging Roles for Transcription Cofactors. Adv. Immunol..

[17] Ikeda N., Yamaguchi Y., Kanaoka M., Ototake Y., Akita A., Watanabe T., Aihara M. (2020). Clinical Significance of Serum Levels of Anti-Transcriptional Intermediary Factor 1-γ Antibody in Patients with Dermatomyositis. J. Dermatol..

[18] Uhlen M., Zhang C., Lee S., Sjöstedt E., Fagerberg L., Bidkhori G., Benfeitas R., Arif M., Liu Z., Edfors F. (2017). A Pathology Atlas of the Human Cancer Transcriptome. Science.

[19] Wang T.Y., Liu Q., Ren Y., Alam S.K., Wang L., Zhu Z., Hoeppner L.H., Dehm S.M., Cao Q., Yang R. (2021). A Pan-Cancer Transcriptome Analysis of Exitron Splicing Identifies Novel Cancer Driver Genes and Neoepitopes. Mol. Cell.

[20] Tsimberidou A.M., Fountzilas E., Bleris L., Kurzrock R. (2022). Transcriptomics and Solid Tumors: The next Frontier in Precision Cancer Medicine. Semin. Cancer Biol..

[21] Peng Q.L., Zhang Y.M., Yang H.B., Shu X.M., Lu X., Wang G.C. (2016). Transcriptomic Profiling of Long Non-Coding RNAs in Dermatomyositis by Microarray Analysis. Sci. Rep..

[22] Gao S., Zhang H., Zuo X., Xiao Y., Liu D., Zhu H., Luo H. (2019). Integrated Comparison of the MiRNAome and MRNAome in Muscles of Dermatomyositis and Polymyositis Reveals Common and Specific MiRNA-MRNAs. Epigenomics.

[23] Yates A.D., Achuthan P., Akanni W., Allen J., Allen J., Alvarez-Jarreta J., Amode M.R., Armean I.M., Azov A.G., Bennett R. (2019). Ensembl 2020. Nucleic Acids Res..

[24] Stelzer G., Rosen N., Plaschkes I., Zimmerman S., Twik M., Fishilevich S., Iny Stein T., Nudel R., Lieder I., Mazor Y. (2016). The GeneCards Suite: From Gene Data Mining to Disease Genome Sequence Analyses. Curr. Protoc. Bioinform..

[25] Karczewski K.J., Francioli L.C., Tiao G., Cummings B.B., Alföldi J., Wang Q., Collins R.L., Laricchia K.M., Ganna A., Birnbaum D.P. (2020). The Mutational Constraint Spectrum Quantified from Variation in 141,456 Humans. Nature.

[26] Lin Y., Liu T., Cui T., Wang Z., Zhang Y., Tan P., Huang Y., Yu J., Wang D. (2020). RNAInter in 2020: RNA Interactome Repository with Increased Coverage and Annotation. Nucleic Acids Res..

[27] Jenjaroenpun P., Wongsurawat T., Yenamandra S.P., Kuznetsov V.A. (2015). QmRLFS-Finder: A Model, Web Server and Stand-Alone Tool for Prediction and Analysis of R-Loop Forming Sequences: Table 1. Nucleic Acids Res..

[28] Scalzitti N., Kress A., Orhand R., Weber T., Moulinier L., Jeannin-Girardon A., Collet P., Poch O., Thompson J.D. (2021). Spliceator: Multi-Species Splice Site Prediction Using Convolutional Neural Networks. BMC Bioinform..

[29] Kyrchanova O., Georgiev P. (2021). Mechanisms of Enhancer-Promoter Interactions in Higher Eukaryotes. Int. J. Mol. Sci..

[30] Petermann E., Lan L., Zou L. (2022). Sources, Resolution and Physiological Relevance of R-Loops and RNA–DNA Hybrids. Nat. Rev. Mol. Cell Biol..

[31] Elsakrmy N., Cui H. (2023). R-Loops and R-Loop-Binding Proteins in Cancer Progression and Drug Resistance. Int. J. Mol. Sci..

[32] Catalanotto C., Cogoni C., Zardo G. (2016). MicroRNA in Control of Gene Expression: An Overview of Nuclear Functions. Int. J. Mol. Sci..

[33] Kazimierczyk M., Kasprowicz M.K., Kasprzyk M.E., Wrzesinski J. (2020). Human Long Noncoding RNA Interactome: Detection, Characterization and Function. Int. J. Mol. Sci..

[34] Huang P., Tang L., Zhang L., Ren Y., Peng H., Xiao Y., Xu J., Mao D., Liu L., Liu L. (2022). Identification of Biomarkers Associated with CD4+ T-Cell Infiltration with Gene Coexpression Network in Dermatomyositis. Front. Immunol..

[35] Franco C., Giannella A., Gasparotto M., Zanatta E., Ghirardello A., Pettorossi F., Rahmè Z., Depascale R., Ragno D., Bevilacqua G. (2024). Circulating Extracellular Vesicles and Small Non-Coding RNAs Cargo in Idiopathic Inflammatory Myopathies Reveal Differences across Myositis Subsets. J. Autoimmun..

[36] Li L., Zuo X., Liu D., Luo H., Zhang H., Peng Q., Wang G., Zhu H. (2022). Plasma Exosomal RNAs Have Potential as Both Clinical Biomarkers and Therapeutic Targets of Dermatomyositis. Rheumatology.

[37] Hirai T., Ikeda K., Tsushima H., Fujishiro M., Hayakawa K., Yoshida Y., Morimoto S., Yamaji K., Takasaki Y., Takamori K. (2018). Circulating Plasma MicroRNA Profiling in Patients with Polymyositis/Dermatomyositis before and after Treatment: MiRNA May Be Associated with Polymyositis/Dermatomyositis. Inflamm. Regen..

[38] Muñoz-Braceras S., Pinal-Fernandez I., Casal-Dominguez M., Pak K., Milisenda J.C., Lu S., Gadina M., Naz F., Gutierrez-Cruz G., Dell’Orso S. (2023). Identification of Unique MicroRNA Profiles in Different Types of Idiopathic Inflammatory Myopathy. Cells.

[39] Georgantas R.W., Streicher K., Greenberg S.A., Greenlees L.M., Zhu W., Brohawn P.Z., Higgs B.W., Czapiga M., Morehouse C.A., Amato A. (2014). Inhibition of Myogenic MicroRNAs 1, 133, and 206 by Inflammatory Cytokines Links Inflammation and Muscle Degeneration in Adult Inflammatory Myopathies. Arthritis Rheumatol..

[40] Pinal-Fernandez I., Casal-Dominguez M., Mammen A.L. (2018). Immune-Mediated Necrotizing Myopathy. Curr. Rheumatol. Rep..

[41] Jain S., Singhal S., Francis F., Hajdu C., Wang J.H., Suriawinata A., Wang Y.Q., Zhang M., Weinshel E.H., Francois F. (2011). Association of Overexpression of TIF1γ with Colorectal Carcinogenesis and Advanced Colorectal Adenocarcinoma. World J. Gastroenterol..

[42] Ding Z.Y., Jin G.N., Wang W., Chen W.X., Wu Y.H., Ai X., Chen L., Zhang W.G., Liang H.F., Laurence A. (2014). Reduced Expression of Transcriptional Intermediary Factor 1 Gamma Promotes Metastasis and Indicates Poor Prognosis of Hepatocellular Carcinoma. Hepatology.

[43] Kassem L., Deygas M., Fattet L., Lopez J., Goulvent T., Lavergne E., Chabaud S., Carrabin N., Chopin N., Bachelot T. (2015). TIF1γ Interferes with TGFβ1/SMAD4 Signaling to Promote Poor Outcome in Operable Breast Cancer Patients. BMC Cancer.

[44] Kim Y., Song K.S., Sohn E.H., Kang S.W., Yoo I.S., Shim S.C., Yoo S.J., Kim J. (2019). Anti-TIF1γ Antibody and the Expression of TIF1γ in Idiopathic Inflammatory Myopathies. Int. J. Rheum. Dis..

[45] Harada Y., Tominaga M., Iitoh E., Kaieda S., Koga T., Fujimoto K., Chikasue T., Obara H., Kakuma T., Ida H. (2022). Clinical Characteristics of Anti-TIF-1γ Antibody-Positive Dermatomyositis Associated with Malignancy. J. Clin. Med..

[46] Scholtissek B., Ferring-Schmitt S., Maier J., Wenzel J. (2017). Expression of the Autoantigen TRIM33/TIF1γ in Skin and Muscle of Patients with Dermatomyositis Is Upregulated, Together with Markers of Cellular Stress. Clin. Exp. Dermatol..

[47] Parkes J.E., Rothwell S., Oldroyd A., Chinoy H., Lamb J.A., Lundberg I.E., Miller F.W., Cooper R.G., Ollier W.E., Gregersen P.K. (2018). Genetic Background May Contribute to the Latitude-Dependent Prevalence of Dermatomyositis and Anti-TIF1-γ Autoantibodies in Adult Patients with Myositis. Arthritis Res. Ther..

[48] Mohassel P., Rosen P., Casciola-Rosen L., Pak K., Mammen A.L. (2015). Expression of the Dermatomyositis Autoantigen Transcription Intermediary Factor 1γ in Regenerating Muscle. Arthritis Rheumatol..

[49] Li C.H., Chen Y. (2016). Insight into the Role of Long Noncoding RNA in Cancer Development and Progression. Int. Rev. Cell Mol. Biol..

[50] Wu G.C., Pan H.F., Leng R.X., Wang D.G., Li X.P., Li X.M., Ye D.Q. (2015). Emerging Role of Long Noncoding RNAs in Autoimmune Diseases. Autoimmun. Rev..

[51] Ye L., Zuo Y., Yang H., Li W., Peng Q., Lu X., Wang G., Shu X. (2019). Specific Autoantibodies and Clinical Phenotypes Correlate with the Aberrant Expression of Immune-Related MicroRNAs in Dermatomyositis. J. Immunol. Res..

[52] El-Sheikh N.M., Abulsoud A.I., Wasfey E.F., Hamdy N.M. (2022). Insights on the Potential Oncogenic Impact of Long Non-Coding RNA Nicotinamide Nucleotide Transhydrogenase Antisense RNA 1 in Different Cancer Types; Integrating Pathway(s) and Clinical Outcome(s) Association. Pathol. Res. Pract..

[53] Lu Q., Liu L., Wang S., Zhang Q., Li L. (2022). Comprehensive Analysis of M5C-Related LncRNAs in the Prognosis and Immune Landscape of Hepatocellular Carcinoma. Front. Genet..

[54] Zhang H.-Y., Yang W., Zheng F.-S., Wang Y., Lu J.-B. (2017). Long Non-Coding RNA SNHG1 Regulates Zinc Finger E-Box Binding Homeobox 1 Expression by Interacting with TAp63 and Promotes Cell Metastasis and Invasion in Lung Squamous Cell Carcinoma. Biomed. Pharmacother..

[55] Alsaleem M.A., Ball G., Toss M.S., Raafat S., Aleskandarany M., Joseph C., Ogden A., Bhattarai S., Rida P.C.G., Khani F. (2020). A Novel Prognostic Two-Gene Signature for Triple Negative Breast Cancer. Mod. Pathol..

[56] Liu Y., Liu X., Lin C., Jia X., Zhu H., Song J., Zhang Y. (2021). Noncoding RNAs Regulate Alternative Splicing in Cancer. J. Exp. Clin. Cancer Res..

[57] Ren P., Lu L., Cai S., Chen J., Lin W., Han F. (2021). Alternative Splicing: A New Cause and Potential Therapeutic Target in Autoimmune Disease. Front. Immunol..

[58] Grabski D.F., Broseus L., Kumari B., Rekosh D., Hammarskjold M.L., Ritchie W. (2021). Intron Retention and Its Impact on Gene Expression and Protein Diversity: A Review and a Practical Guide. Wiley Interdiscip. Rev. RNA.

[59] Greenberg S.A. (2010). Dermatomyositis and Type 1 Interferons. Curr. Rheumatol. Rep..

[60] Leylek R., Idoyaga J. (2019). The Versatile Plasmacytoid Dendritic Cell: Function, Heterogeneity, and Plasticity. Int. Rev. Cell Mol. Biol..

[61] Sharma S. (2017). Immunomodulation: A Definitive Role of MicroRNA-142. Dev. Comp. Immunol..

[62] Shrestha A., Mukhametshina R.T., Taghizadeh S., Vásquez-Pacheco E., Cabrera-Fuentes H., Rizvanov A., Mari B., Carraro G., Bellusci S. (2017). MicroRNA-142 Is a Multifaceted Regulator in Organogenesis, Homeostasis, and Disease. Dev. Dyn..

[63] Kinder T.B., Heier C.R., Tully C.B., Van der Muelen J.H., Hoffman E.P., Nagaraju K., Fiorillo A.A. (2020). Muscle Weakness in Myositis: MicroRNA-Mediated Dystrophin Reduction in a Myositis Mouse Model and Human Muscle Biopsies. Arthritis Rheumatol..

[64] Li L., Zuo X., Liu D., Luo H., Zhu H. (2021). The Functional Roles of RNAs Cargoes Released by Neutrophil-Derived Exosomes in Dermatomyositis. Front. Pharmacol..

[65] Xue Y. (2022). Architecture of RNA–RNA Interactions. Curr. Opin. Genet. Dev..

