# Supplementary material for: Sequence Alignment between TRIM33 Gene and Human Noncoding RNAs: A Potential Explanation for Paraneoplastic Dermatomyositis
Source: J Pers Med. 2024 Jun 13;14(6):628. doi: 10.3390/jpm14060628 (PMC11204533; doi:10.3390/jpm14060628)
Supplement: Supplementary file 1 [file jpm-14-00628-s001.zip › FIGURE S1 SPLICEATOR ANALYSIS.pdf]

SPLICEATOR ANALYSIS

Reliability**:** Donor 98%; Acceptor 98%

Model: 200

Splice site: Both

TRIM33 INTRON 1

FROM 9987 TO 10297 bp

aattttttttttttttttttt**gaga**cggagtctcgctctgtcgcccaggctggagtgcagtggcggatctcggctcact
gcaagctccgcctcccgggttcacgccattctcctgcctcagcctcccaagtagctgggactacaggcgcccgccactac
gcccggctaatttttgtatttttagtagagacggggttttaccgtgttagccgggatggtctcgatctcctgacctcgt
gatccgcccgcctcggcctcccaaagtgctgggattacaggcgtgagccaccgcgcccggcccc

Splicing Site	Position	Score	Sequence
Acceptor #1	21	0.983	TTTT**G**AGACG
Acceptor #2	22	0.989	TTTGA**G**ACGG
Acceptor #3	23	0.985	TTGA**GA**CGGA

FROM 23274 TO 23593 bp

tttttttttttttttttttttttttt**tgag**acggagtctcgctctgtcgcccaggccggactgcggactgcagtggcgca
atctcggctcattgcaagctccgcttcccgggttcacgccattctcctgcctcagcctcccgagtagctgggactacagg
cgcccgccaccgcgcccggctaatttttgtatttttagtagagacggggtttcaccttgttagccaggatggtctcgat
ctcctgacctcatgatccacccgcctcggcctcccaaagtgctgggattacaggcgtgagccaccgcgcccggcctattt

Splicing Site	Position	Score	Sequence
Acceptor #1	26	0.986	TTTT**TG**AGAC
Acceptor #2	27	0.992	TTTT**G**AGACG
Acceptor #3	28	0.991	TTTGA**G**ACGG
Acceptor #4	29	0.988	TTGA**GA**CGGA

FROM 30709 TO 31395 bp

Ttttttttttttttttttaaattgatcattcttgggtgtttctcgc**ag**agggggatttggcagggtcataggacaatag
tggaggaaggtcagcagataaacaagtgaacaaaggtctctggttttcctaggcagaggaccctgcggccttccgaagtg
tttgtgtccctgggtacttgagatcagggagtggtgatgactcttaaggagcatactgccttcaagcatctgtttaacaa
agcacatcttgcaccgcccttaatccatttaaccctgagtggacacagcacatgtttcagagagcacagggttgggggca
aggtcatagatcaacagcatcccaaggcagaagaatctttcttagtacagaacaaaaatggagtctcctatgtctacttc
tttctacacagacacagcaacgatctgatttctctatctttccccacatttccccctttttctattcca**ca**aaaccgccat
catcatcatggcctgttctcaatgagctgttgggtacacctcccagacggggtggcggccgggcagaggggctcctcact
tcccagcaggggcggccgggcagaggcgcccccaccttccggatggggcggcggccgggtggaggcgccccccttcctccc
tcccggacggggcagctggccgggcgggggctgccccccacctccct

Splicing Site	Position	Score	Sequence
Acceptor #1	47	0.983	TCGC**AG**AGGG
Acceptor #2	468	0.982	TCCA**CA**AAAC
