# Supplementary material for: Sequence Alignment between TRIM33 Gene and Human Noncoding RNAs: A Potential Explanation for Paraneoplastic Dermatomyositis
Source: J Pers Med. 2024 Jun 13;14(6):628. doi: 10.3390/jpm14060628 (PMC11204533; doi:10.3390/jpm14060628)

ENST00000358465

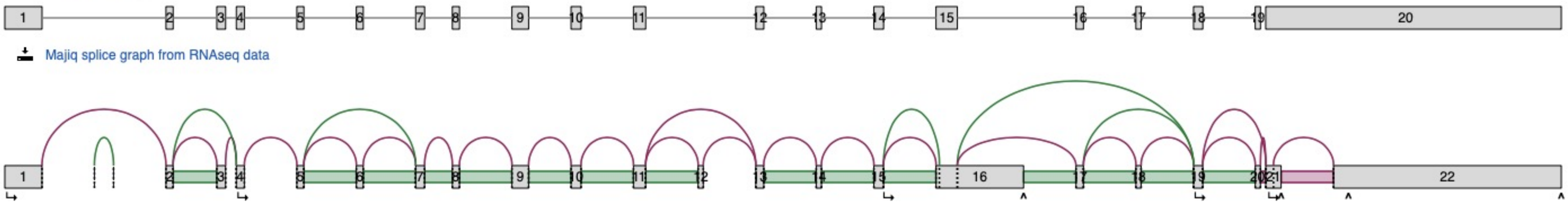

ENST00000369543

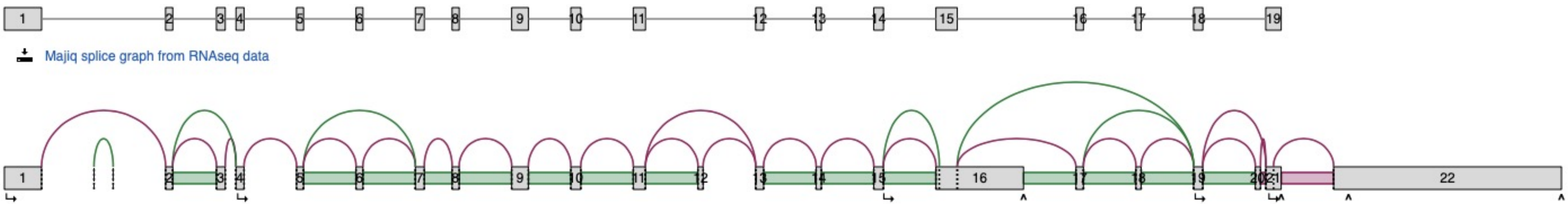

ENST00000448034

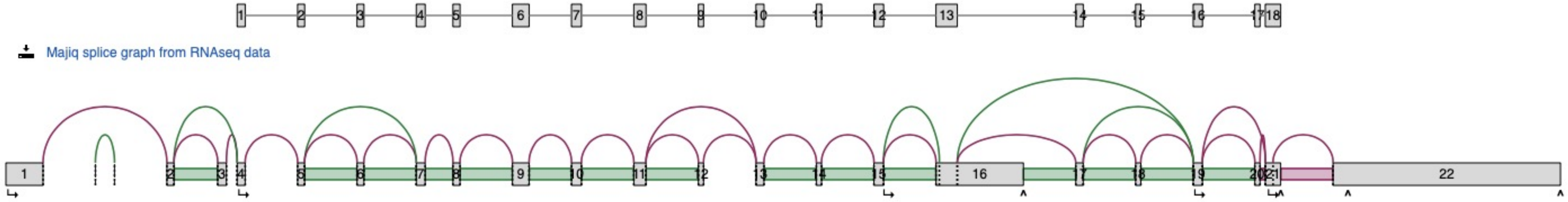

ENST00000476908

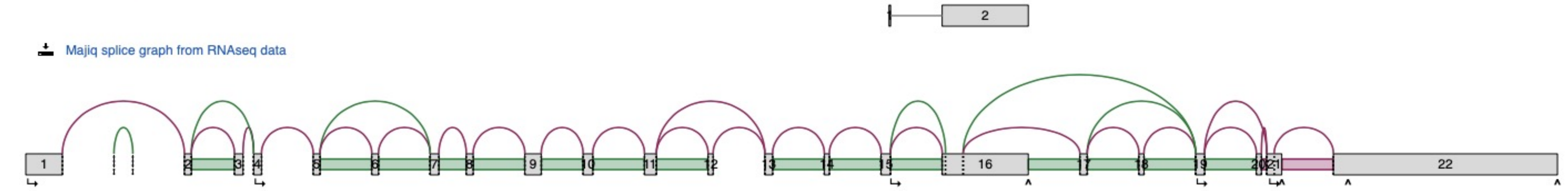

ENST00000478032

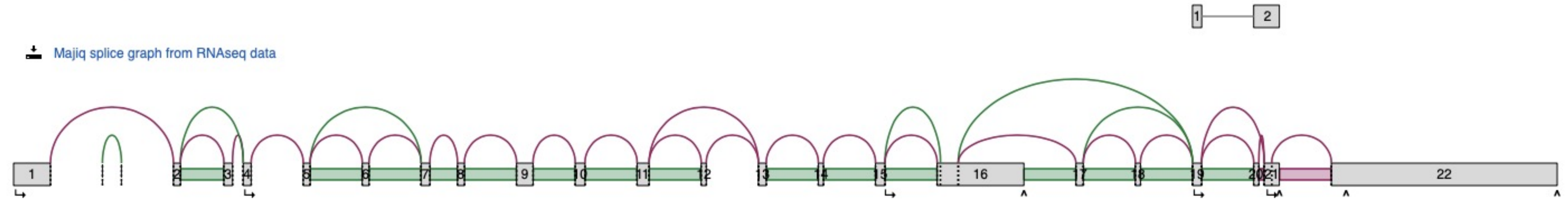

ENST00000492227

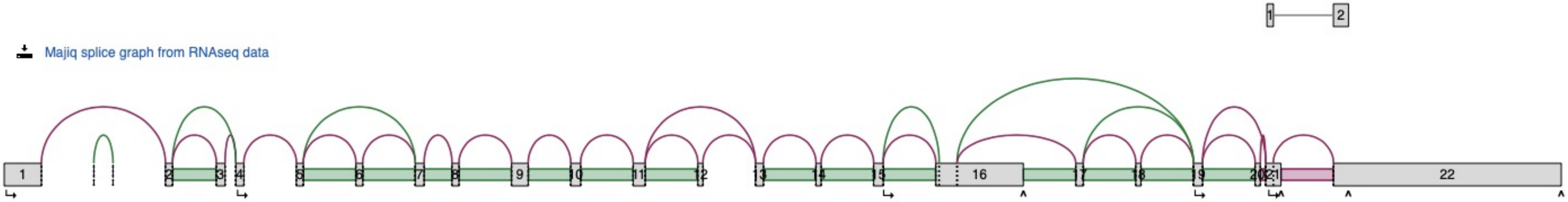

Supplement: Supplementary file 1 [file jpm-14-00628-s001.zip › figure S2 TRIM REV.pdf]
